# Integrated metabolic profiling and transcriptome analysis of pigment accumulation in *Lonicera japonica* flower petals during colour-transition

**DOI:** 10.1186/s12870-021-02877-y

**Published:** 2021-02-17

**Authors:** Yan Xia, Weiwei Chen, Weibo Xiang, Dan Wang, Baogui Xue, Xinya Liu, Lehua Xing, Di Wu, Shuming Wang, Qigao Guo, Guolu Liang

**Affiliations:** 1 Key Laboratory of Horticulture Science for Southern Mountains Regions of Ministry of Education; College of Horticulture and Landscape Architecture, Southwest University, Chongqing, 400715 China; 2 Academy of Agricultural Sciences of Southwest University, State Cultivation Base of Crop Stress Biology for Southern Mountainous Land of Southwest University, Chongqing, 400715 China; 3 Henan International Joint Laboratory of Crop Gene Resources and Improvement, School of Agricultural Sciences, Zhengzhou University, Zhengzhou, 450001 Henan China; 4 Rare Plant Research Institute of the Yangtze River (Yichang); Institute of Science and Technology, China Three Gorges Corporation, Beijing, 100083 China

**Keywords:** *Lonicera japonica*, Petal colour, Pigment, Gene expression, Endogenous hormones

## Abstract

**Background:**

Plants have remarkable diversity in petal colour through the biosynthesis and accumulation of various pigments. To better understand the mechanisms regulating petal pigmentation in *Lonicera japonica*, we used multiple approaches to investigate the changes in carotenoids, anthocyanins, endogenous hormones and gene expression dynamics during petal colour transitions, i.e., green bud petals (GB_Pe), white flower petals (WF_Pe) and yellow flower petals (YF_Pe).

**Results:**

Metabolome analysis showed that YF_Pe contained a much higher content of carotenoids than GB_Pe and WF_Pe, with α-carotene, zeaxanthin, violaxanthin and γ-carotene identified as the major carotenoid compounds in YF_Pe. Comparative transcriptome analysis revealed that the key differentially expressed genes (DEGs) involved in carotenoid biosynthesis, such as *phytoene synthase*, *phytoene desaturase* and *ζ-carotene desaturase*, were significantly upregulated in YF_Pe. The results indicated that upregulated carotenoid concentrations and carotenoid biosynthesis-related genes predominantly promote colour transition. Meanwhile, two anthocyanins (pelargonidin and cyanidin) were significantly increased in YF_Pe, and the expression level of an *anthocyanidin synthase* gene was significantly upregulated, suggesting that anthocyanins may contribute to vivid yellow colour in YF_Pe. Furthermore, analyses of changes in indoleacetic acid, zeatin riboside, gibberellic acid, brassinosteroid (BR), methyl jasmonate and abscisic acid (ABA) levels indicated that colour transitions are regulated by endogenous hormones. The DEGs involved in the auxin, cytokinin, gibberellin, BR, jasmonic acid and ABA signalling pathways were enriched and associated with petal colour transitions.

**Conclusion:**

Our results provide global insight into the pigment accumulation and the regulatory mechanisms underlying petal colour transitions during the flower development process in *L. japonica*.

**Supplementary Information:**

The online version contains supplementary material available at 10.1186/s12870-021-02877-y.

## Background

Different plant lineages have adopted various mechanisms of flower colour determination to fulfil the requirements for pollinator attraction or non-pollinator-related traits [1]. Due to the importance of colour formation in angiosperms, especially in ornamentals, the biosynthesis pathways of pigments in colour formation have been extensively reported [2–6]. Three major classes of pigments, flavonoids/anthocyanins, carotenoids and chlorophylls, are distributed ubiquitously in plants. Among them, pigmented flavonoids, mainly anthocyanins, are major pigmentation compounds in flowering plants [5]. Carotenoids also widely participate in the yellow-to-red colouration of flowers [7]. Chlorophylls, which are a class of essential photosynthetic components, exist in almost all plants and are mainly involved in the formation of green colour in flowers [8]. Moreover, anthocyanins and carotenoids often coexist simultaneously, and their combination causes diversity in flower colour [9].

As one of the main subgroups of flavonoids, anthocyanins primarily cause the colour formation of red, orange, blue and purple flower colours [5]. The specific colouration of anthocyanin is also greatly dependent on various moieties, co-pigments and pH [9]. Anthocyanins in plants are synthesized via the flavonoid pathway, and their biosynthetic pathway and genes have been well characterized [5, 9, 10]. It was reported that anthocyanin synthesis shares the same upstream pathway as the formation of anthocyanidins (cyanidin, delphinidin, and pelargonidin), followed by specific downstream branch for anthocyanin modification. In this comprehensive synthesis process, the formation of anthocyanidins by the catalysis of anthocyanidin synthase (ANS; also known as leucoanthocyanidin dioxygenase, LDOX) is an important node that leads to flavonoid flux into the anthocyanin branch.

Carotenoids, a kind of C_40_ isoprenoids, are distributed in some flowers and provide distinct colours ranging from yellow/orange to red [3, 4]. In the initial steps of the carotenoid biosynthetic pathway, the key enzymes have been well characterized, including phytoene synthase (PSY), phytoene desaturase (PDS) and ζ-carotene desaturase (ZDS) [11–13]. Although the biosynthetic pathways of anthocyanins and carotenoids are well established, the balance of the expression dynamics of anthocyanins and carotenoids-related genes during petal colour transition in a single flower remains poorly understood.

Changes in flower colour are comprehensively regulated by physiological changes and transcriptional level fluctuations in related genes. To date, high-resolution mass spectrometry (MS)-based metabolomics represents an effective technique to detect the accumulation and dynamic changes of metabolites [14–16]. Furthermore, transcriptome analysis has developed into a powerful approach that provides abundant sequence resources to study the mechanisms regulating flower colour formation [17, 18]. To identify pigment accumulation, endogenous hormone changes and related gene fluctuations in petal colour transitions, global analysis of the metabolome combined with the transcriptional levels of pigment biosynthesis genes is required.

*Lonicera japonica* Thunb. is known as “gold and silver flower” in China and is widely cultivated in East Asian countries [19, 20]. It has excellent ornamental properties due to the dynamic petal colours of every single flower and provides plant materials to uncover the molecular mechanisms of petal colour transition [21–24]. In our study, we reported the metabolic profiling of carotenoids, anthocyanins, endogenous hormones and gene expression dynamics in *L. japonica* petals at various stages (i.e., green flower bud, white flower and yellow flower) using integrated analyses of the metabolome, physiology, and transcriptome. With this extensive analysis of multiple data points in *L. japonica* petals, we reveal changes in the key pigments, hormones, and related biosynthesis genes that are associated with petal colour transitions.

## Methods

### Plant materials sampling and color detection

According to a previous study [21], flower buds and opening flowers of *Lonicera japonica* Thunb. were collected from the Beijing Botanical Garden, Beijing, China, with permission. The colour of buds/flowers gradually changed from green to white and then to yellow during floral development. Three developmental stages of petal, i.e., green bud petals (GB_Pe), white flower petals (WF_Pe) and yellow flower petals (YF_Pe), were selected to perform pigment metabolome, transcriptome and plant hormone analyses. For each biological replication, petals were dissected from 3 to 6 uniform flowers, weighed, sampled, frozen immediately in liquid nitrogen and stored at − 80 °C until use. Meanwhile, the colour index of petals was measured using a CR-400 chroma meter (Konica Minolta Sensing Inc., Osaka, Japan). Hunter parameters of L*, a* and b* were mainly used according to the CIELAB colour model.

### Carotenoid extraction and quantification

Petal samples from GB_Pe, WF_Pe and YF_Pe were used for carotenoid extraction. Three biological replicates were performed for each developmental stage. Each replicate (~ 1 g of fresh weight) was freeze-dried and ground into fine powder, and then about 100 mg of powder was dissolved in 1 mL of a solution of n-hexane: acetone: ethanol (2:1:1 (V:V:V)). The solution sample was vortexed (30 s), followed by ultrasound-assisted extraction at room temperature for 20 min, and centrifuged at 12,000 g for 5 min to collect the supernatant. The extraction steps above were repeated, and the supernatants from the two centrifugations were combined. Subsequently, the combined supernatant was evaporated to dryness using a nitrogen gas stream and then reconstituted in 200 μL of (acetonitrile:methanol = 3:1 (V:V)): methyl tert-butyl ether = 85:15 (V:V). Finally, the solution was centrifuged at 12,000 g for 2 min to collect the supernatant for LC-MS/MS analysis. To monitor the stability of the LC-MS/MS analytical conditions, a quality control (QC) sample was used with equal mixing of all measured samples and was run at intervals during the assay. Stock solutions of standards purchased from Sigma-Aldrich (St. Louis, MO, USA) or Olchemim Ltd. (Olomouc, Czech Republic) were prepared at a concentration of 10 mg/mL in HPLC-grade acetonitrile (ACN) and then diluted with ACN to working solutions. For each carotenoid, five successive concentration gradients were used to plot the standard curve.

The prepared sample solutions were analysed by an LC-APCI-MS/MS system (UPLC, Shim-pack UFLC SHIMADZU CBM30A system, www.shimadzu.com.cn/; MS, Applied Biosystems 6500 Triple Quadrupole, www.appliedbiosystems.com.cn/) equipped with an APCI Turbo Ion-Spray interface and operated in positive ion mode and controlled by Analyst 1.6.3 software (Applied Biosystems Company, Framingham, MA, USA) at Wuhan Metware Biotechnology Co., Ltd. (Wuhan, China). The APCI source operation parameter settings and multiple reaction monitoring (MRM) transitions were performed according to a previous study [25]. Finally, the carotenoid levels were calculated according to the following formula: carotenoid content (μg/g) = A*(B/1000)/C (A: the carotenoid concentration calculated by the standard curve using chromatographic peak area integrals, μg/mL; B: the volume of solution for redissolution, μL; C: the dry weight of plant tissue powder, g). SPSS 16.0 was used for the analysis of variance (ANOVA). The differences in means were compared using Duncan’s multiple range test.

### Anthocyanin extraction and analyses

Nine petal samples (each sample was parallel to the above sample in the carotenoid analyses) were used for anthocyanin analyses. The freeze-dried petals were crushed into powder by a mixer mill (MM 400, Retsch). Each powder sample weighing ~ 100 mg was then extracted overnight at 4 °C with 70% (V/V) aqueous methanol and centrifuged at 10,000 g for 10 min. The supernatant extract was absorbed using a CNWBOND Carbon-GCB SPE Cartridge (250 mg, 3 mL; ANPEL, Shanghai, China) and filtered through a 0.22 μm SCAA-104 membrane (ANPEL, Shanghai, China). To examine the precision and repeatability of the instrumental assay system and analysis process, the QC sample was prepared by blending all of the samples equally and inserting test samples at intervals. The prepared samples were analysed by an LC-ESI-MS/MS system at Wuhan Metware Biotechnology Co., Ltd. The HPLC conditions and ESI source operation parameters were set according to previous studies [26, 27].

Qualitative and quantitative analyses of anthocyanins were basically consistent with the analyses of carotenoids above. Specifically, anthocyanin identification was performed based on the MWDB database (Wuhan MetWare Biotechnology Co., Ltd.) and publicly available metabolite databases following standard procedures if the standards were unavailable. Anthocyanin with a statistically significant difference in content was determined with thresholds of fold change ≥1.6 or ≤ 0.625, *P*-value < 0.05, and variable importance in projection (VIP) ≥ 0.8.

### Determination of various hormones during flower development

The contents of indoleacetic acid (IAA), zeatin riboside (ZR), gibberellic acid (GA_3_), brassinosteroid (BR), methyl jasmonate (MeJA) and abscisic acid (ABA) from GB_Pe, WF_Pe and YF_Pe were measured by an indirect ELISA technique. The extraction, purification and determination of each hormone were performed according to previous study [28, 29] and the instructions of the corresponding kit produced by Shanghai Enzyme-linked Biotechnology Co., Ltd. (Shanghai, China). Each stage was prepared with three biological replicates. All data were analysed by ANOVA using SPSS 16.0, and the differences in means were compared by Duncan’s multiple range test.

### RNA isolation, cDNA library construction and sequencing

Petal samples of GB_Pe, WF_Pe and YF_Pe were collected and three independent biological replicates were used. Total RNA was extracted from plant tissue using TRIzol reagent (Invitrogen, Carlsbad, CA, USA) following the manufacturer’s protocol and digested with DNase I (Takara, Dalian, China). The quality and purity of total RNA were evaluated by stringent RNA quality control. cDNA library construction and sequencing were performed by Annoroad Gene Technology (Beijing, China). Each constructed cDNA library (∼10 ng) was subjected to paired-end 150 bp sequencing on an Illumina HiSeq™ 4000 system (San Diego, CA, USA) according to the manufacturer’s instructions.

### Data assembly and annotation

The raw reads were filtered to remove adapter-polluted reads, low-quality reads, and reads with more than 5% ambiguous nucleotides. These clean reads with high-quality were subjected to the following analyses. Trinity software [30] was used to perform the de novo transcriptome assembly with default parameter values.

The assembled unigenes were annotated by homology search to publicly accessible databases using local BLAST programmes (version 2.2.28) with a significance threshold of E < 1e-5. Meanwhile, all unigenes were analysed with Blast2GO (version 3.0.8) to obtain the gene ontology (GO) annotations [31], which included BP, CC, and MF, with an *E*-value cut-off = 1e-5. Web Gene Ontology annotation software was adopted to perform GO functional classifications [32]. Furthermore, the sequences were searched against the Kyoto Encyclopedia of Genes and Genomes (KEGG) database using KEGG Automatic Annotation Server (KAAS) with an E-value threshold <1e-10.

### Differentially expressed gene (DEG) identification and analysis

The expression level of each unigene was calculated by reads per kilobase millon mapped reads (RPKM) to assess the length and depth of sequencing [33]. Then, the differences in the expression abundance of each gene between each pair of compared samples were calculated by DESeq 2 software (version 1.4.5) [34]. Each resulting *p*-value was adjusted to a *q*-value, following the Benjamini-Hochberg procedure for controlling the false discovery rate [35]. The DEGs were identified with *q* ≤ 0.05 and |log2(fold-change)| ≥ 1 as thresholds. Then, the GO and KEGG analyses were considered to be significantly enriched with *q* ≤ 0.05 [36]. A GO functional enrichment analysis was performed using the BiNGO plugin of Cytoscape [37].

### Reverse transcription quantitative PCR (RT-qPCR) analysis

Two micrograms of total RNA from *L. japonica* petals was reverse transcribed using the M-MuLV Reverse Transcriptase Kit (Takara, Japan) and oligo (dT) primers according to the manufacturer’s instructions. The diluted cDNA reaction mixture was used as a template in a 20 μl PCR for transcript measurements. qPCR was carried out in a Bio-Rad CFX96™ Real-Time System. The qPCR programme was initiated with a preliminary step of 94 °C for 5 min, followed by 45 cycles of 94 °C for 10 s, 56 °C for 10 s, and 72 °C for 10 s. A melting curve was generated for each sample at the end of each run to ensure the purity of the amplified products with a temperature change of 0.5 °C/s from 65 °C to 95 °C and 5 s for each step afterwards for the melt curve. For each sample, three biological replicates were used. The gene-specific primers of qPCR were designed according to the selected sequences derived from RNA-seq (Table S1). The expression level of *Actin* was applied to normalize the mRNA levels for each sample [21]. All data were analysed by ANOVA, and the mean differences were compared utilizing Duncan’s multiple range test.

## Results

### Morphology analysis of petal colour transitions

**Fig. 1:**
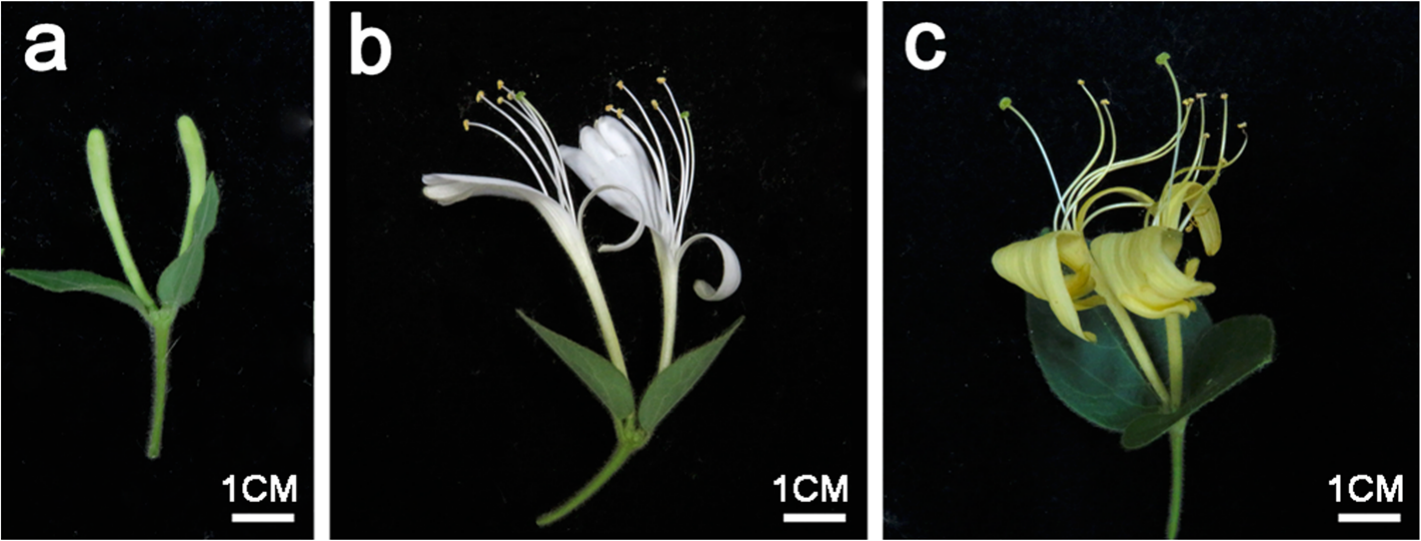
Morphological observation of *L. japonica* flowers. **a** Flower buds with green petals (GB_Pe). **b** Flowers with white petals (WF_Pe). **c** Flowers with yellow petals (YF_Pe)

The petal colour of every single flower was transformed continuously from green to white and then to yellow during flower development in *L. japonica*. Early in the development of floral buds, primary buds with green petals grew to approximately 3.5 cm in length (Fig. 1a). At the early stage of anthesis, the petals turned from green to white (Fig. 1b). Then, the petals gradually transformed to yellow from white before the withering stage (Fig. 1c). During petal colour transitions, petals at the green bud, white flower and yellow flower stages were selected. The changes in the colour index of GB_Pe, WF_Pe and YF_Pe were significantly different (Table S2). The values of redness (a*) in GB_Pe, WF_Pe and YF_Pe were − 12.36, − 0.58 and 1.25, respectively. The parameter lightness (L*) in WF_Pe was 82.40, which was higher than that in GB_Pe and YF_Pe. The index of yellowness (b*) in YF_Pe was the highest (42.55).

### Carotenoid and anthocyanin accumulation in ***L.****japonica* petals at various stages

To obtain an accurate understanding of carotenoid accumulation, carotenoid profiling was analysed in *L. japonica* petals using LC-MS/MS during petal colour transitions. A total of 13 carotenoids were detected from GB_Pe, WF_Pe and YF_Pe (Table 1). The major carotenoids of GB_Pe were lutein, violaxanthin, neoxanthin and zeaxanthin. The lutein content significantly decreased from 39.30 μg/g in GB_Pe to 1.46 μg/g in WF_Pe and slightly decreased to 1.09 μg/g in YF_Pe. The violaxanthin content first significantly decreased from 18.87 μg/g in GB_Pe to 0.60 μg/g in WF_Pe and then drastically increased to 43.81 μg/g in YF_Pe. The trends of neoxanthin and zeaxanthin were similar to those of violaxanthin. Compared with GB_Pe and WF_Pe, most of carotenoids, including α-carotene, antheraxanthin, lycopene, zeaxanthin, violaxanthin, γ-carotene, neoxanthin, β-carotene, β-cryptoxanthin and apocarotenal, were significantly upregulated in YF_Pe. Among them, violaxanthin (43.81 μg/g), zeaxanthin (27.45 μg/g), α-carotene (20.84 μg/g) and γ-carotene (19.97 μg/g) were the major carotenoid compounds in YF_Pe. Except for zeaxanthin, the contents of the remaining 12 carotenoids were all detected at lower levels in WF_Pe.

**Table 1:** Contents (μg/g DW) of carotenoids in GB_Pe, WF_Pe and YF_Pe

Compounds	Molecular Weight (Da)	Q1 (Da)	Q3 (Da)	Rt (min)	GB_Pe	WF_Pe	YF_Pe
α-Carotene	536.438	537.6	123.1	2.6	9.54 ± 0.55^b^	0.00^c^	20.84 ± 0.54^a^
Antheraxanthin	584.423	585.5	175.4	1.0	0.75 ± 0.00^b^	0.75 ± 0.00^b^	1.25 ± 0.04^a^
Lycopene	536.438	537.4	81.0	3.1	0.00^b^	0.00^b^	2.15 ± 0. 02^a^
Zeaxanthin	568.428	569.4	477.5	1.4	13.15 ± 0.70^b^	10.61 ± 0.08^c^	27.45 ± 1.10^a^
Violaxanthin	600.418	601.4	221	0.9	18.87 ± 3.99^b^	0.60 ± 0.02^c^	43.81 ± 0.76^a^
γ-Carotene	536.438	537.6	177.3	3.3	0.00^b^	0.00^b^	19.97 ± 0.97^a^
Neoxanthin	600.418	601.4	565.5	0.6	14.23 ± 1.31^b^	1.14 ± 0.04^c^	18.05 ± 0.21^a^
β-Carotene	536.438	537.6	177.4	3.1	0.00^b^	0.00^b^	1.85 ± 0.03^a^
Lutein	568.428	551.5	175.4	1.0	39.30 ± 2.86^a^	1.46 ± 0.04^b^	1.09 ± 0.04^b^
β-Cryptoxanthin	552.433	553.5	461.5	2.2	0.00^b^	0.00^b^	17.82 ± 0.45^a^
Astaxanthin	596.840	597.4	379.1	0.8	0.00^c^	1.26 ± 0.00^a^	1.21 ± 0.00^b^
Apocarotenal	416.638	417.3	325.1	1.2	1.06 ± 0.00^b^	0.00^c^	1.07 ± 0.00^a^
ε-Carotene	536.438	537.6	123.2	2.2	0.05 ± 0.00^a^	0.00^b^	0.00^b^
Total					96.96	15.82	156.56

Data are expressed as the mean ± SD of three biological replications. Different letters indicate significant differences at *P* < 0.05 (Duncan’s multiple range test)

To better understand the content changes of anthocyanins, quantitative analysis of anthocyanins was further performed by LC-MS/MS technology. A total of 10 anthocyanins were identified from GB_Pe, WF_Pe and YF_Pe (Table 2). In GB_Pe, delphinidin was the predominant component of anthocyanins. Specifically, delphinidin was reduced by 3.55- and 2.85-fold in WF_Pe and YF_Pe, respectively, compared with GB_Pe (Table S3). In contrast, pelargonidin was not detected in GB_Pe but at higher levels in WF_Pe and YF_Pe. In the YF_Pe vs GB_Pe comparison, pelargonidin and cyanidin O-syringic acid significantly increased, while delphinidin, cyanidin O-malonyl-malonylhexoside and delphin chloride significantly decreased. Compared with WF_Pe, the contents of pelargonidin and cyanidin were increased by 2.11- and 2.36-fold in YF_Pe, respectively. However, cyanidin O-malonyl-malonylhexoside and delphin chloride were not detected in YF_Pe.

**Table 2:** Anthocyanins in GB_Pe, WF_Pe and YF_Pe

Compounds	Molecular Weight (Da)	Q1 (Da)	Q3 (Da)	Rt (min)	Peak area		
					GB_Pe	WF_Pe	YF_Pe
Cyanidin O-malonyl-malonylhexoside	621.10	621.1	287.3	3.26	87,500 ± 15,836	80,533 ± 6901	NA
Cyanidin O-syringic acid	466.10	465.1	285.1	2.59	15,367 ± 4716	41,467 ± 3500	51,900 ± 3404
Peonidin O-malonylhexoside	548.10	547.1	503.1	3.00	20,633 ± 839	30,267 ± 6700	28,567 ± 3102
Delphinidin	303.24	303.0	229.0	2.98	13,366,667 ± 611,010	2,960,000 ± 217,945	3,423,333 ± 285,715
Pelargonidin	271.24	271.0	149.0	3.85	NA	628,333 ± 11,930	1,953,333 ± 77,675
Delphinidin 3-O-glucoside	465.10	465.1	303.0	2.26	200,333 ± 2517	193,333 ± 4509	181,333 ± 14,012
Cyanidin 3,5-O-diglucoside	611.00	611.0	287.0	2.08	833,333 ± 13,503	494,333 ± 9609	657,333 ± 17,616
Petunidin 3-O-glucoside	479.00	479.0	317.0	2.56	1,316,667 ± 20,817	1,653,333 ± 28,868	1,676,667 ± 35,119
Cyanidin	287.24	287.0	213.0	3.54	352,333 ± 63,066	245,000 ± 85,282	823,333 ± 47,606
Delphin chloride	662.12	627.2	303.1	1.88	12,633 ± 2857	15,600 ± 2007	NA

The results of the peak area are the mean ± SD of three biological replications

### Effects of endogenous hormones during petal color transitions

**Fig. 2:**
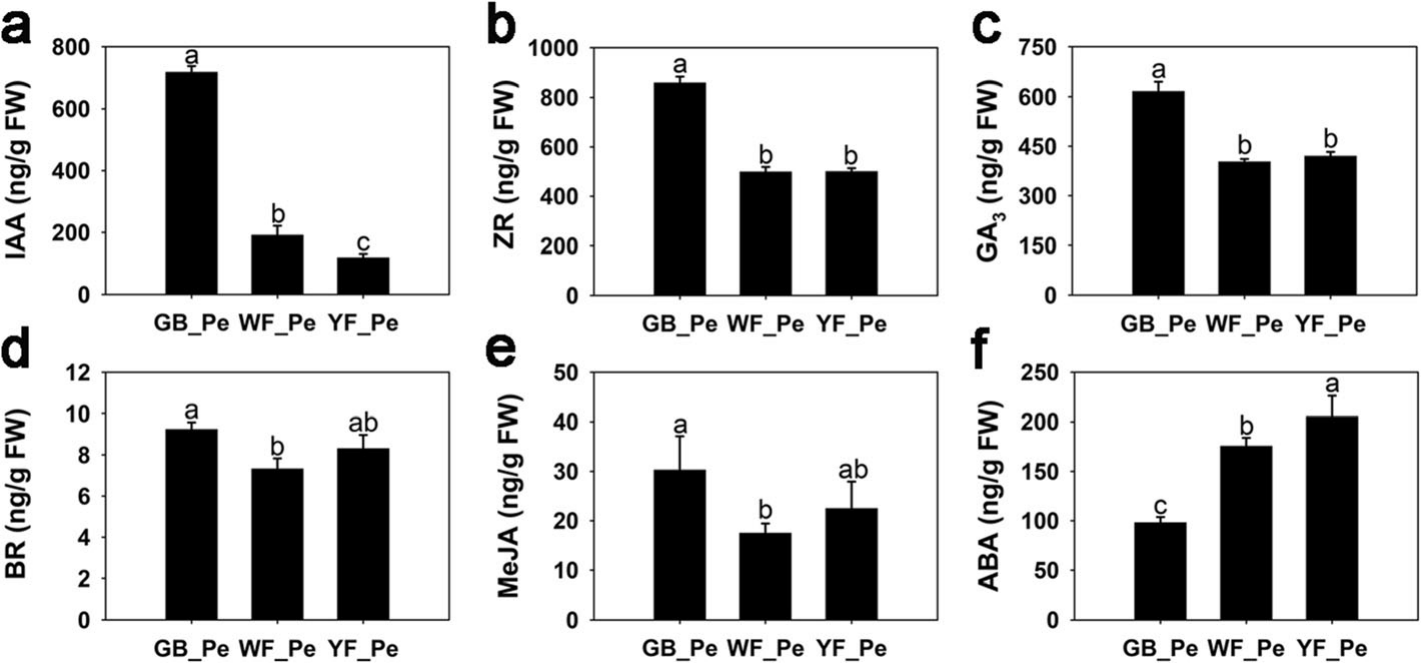
Concentrations of endogenous hormones in petal color transitions in *L. japonica*. **a** IAA concentration. **b** ZR concentration. **c** GA_3_ concentration. **d** BR concentration. **e** MeJA concentration. **f** ABA concentration. Significant differences are indicated by different letters at *P* < 0.05

To obtain the changes in endogenous hormones, the concentrations of IAA, ZR, GA_3_, BR, MeJA and ABA were analysed. During petal colour transitions, the concentrations of IAA, ZR, GA_3_, BR and MeJA decreased, but the content of ABA increased (Fig. 2). The IAA concentration decreased significantly from 717.3 ng·g^− 1^ FW (GB_Pe) to 191.0 ng·g^− 1^ FW (WF_Pe) and then to 118.8 ng·g^− 1^ FW (YF_Pe). The ZR and GA_3_ concentrations both first decreased significantly from GB_Pe to WF_Pe and then remained stable from WF_Pe to YF_Pe. The BR concentration was highest in GB_Pe. From GB_Pe to YF_Pe, the BR concentration decreased significantly from 9.2 ng·g^− 1^ FW in GB_Pe to 7.3 ng·g^− 1^ FW in WF_Pe and then increased slightly to 8.3 ng·g^− 1^ FW in YF_Pe. The level of MeJA first decreased significantly from GB_Pe to WF_Pe, reaching the lowest level, and then slightly increased from WF_Pe to YF_Pe. However, the ABA concentration increased significantly from 98.0 ng·g^− 1^ FW to 205.2 ng·g^− 1^ FW from GB_Pe to YF_Pe (Fig. 2).

### Sequencing, de novo assembly and annotation

To identify key candidate genes for petal colour transitions, RNA sequencing was carried out from GB_Pe, WF_Pe and YF_Pe. Nine cDNA libraries were sequenced and 448,565,884 raw reads were generated. After data filtering, 408,576,816 (91.1%) clean reads were produced, and the *Q*30 values were greater than 96.7%. For each sample, clean reads were obtained from 6.6 to 7.1 Gb (Table S4). A total of 69,946 unigenes were generated with an average length of 871 bp and an N50 of 1636 bp (Table S5). Most unigenes (96.6%) were generated from 200 to 3200 bp in length, and 2383 (3.4%) unigenes were more than 3200 bp in length (Fig. S1).

A total of 34,068 assembled unigenes were annotated (Table S6). Based on sequence similarity, 22,662 (32.4%) unigenes were enriched into three groups (biological process, cellular component and molecular function) based on GO term analysis (Fig. S2). The biological processes were mainly focused on ‘cellular process’ and ‘metabolic process’. The cellular components were mainly involved in ‘cell part’. The molecular functions were mainly classified into ‘binding’ and ‘catalytic activity’. KEGG term analysis was used to identify the functional classifications of the unigenes. A total of 9309 (13.31%) unigenes were enriched in 32 KEGG pathway groups, of which ‘signal transduction’ represented the largest group, followed by ‘carbohydrate metabolism’, ‘translation’ and ‘folding, sorting and degradation’ (Fig. S3).

### Identification and analysis of DEGs

To detect alterations in gene expression, transcriptomic analyses of WF_Pe vs GB_Pe, YF_Pe vs WF_Pe and YF_Pe vs GB_Pe were carried out to identify the key DEGs during petal colour transition in *L. japonica* (Fig. S4). A total of 29,679 DEGs were identified based on a 2-fold change at *P* < 0.05 (Fig. S4a). For each comparison, the numbers of total DEGs, upregulated DEGs and downregulated DEGs were counted, as shown in Fig. S4b.

**Fig. 3:**
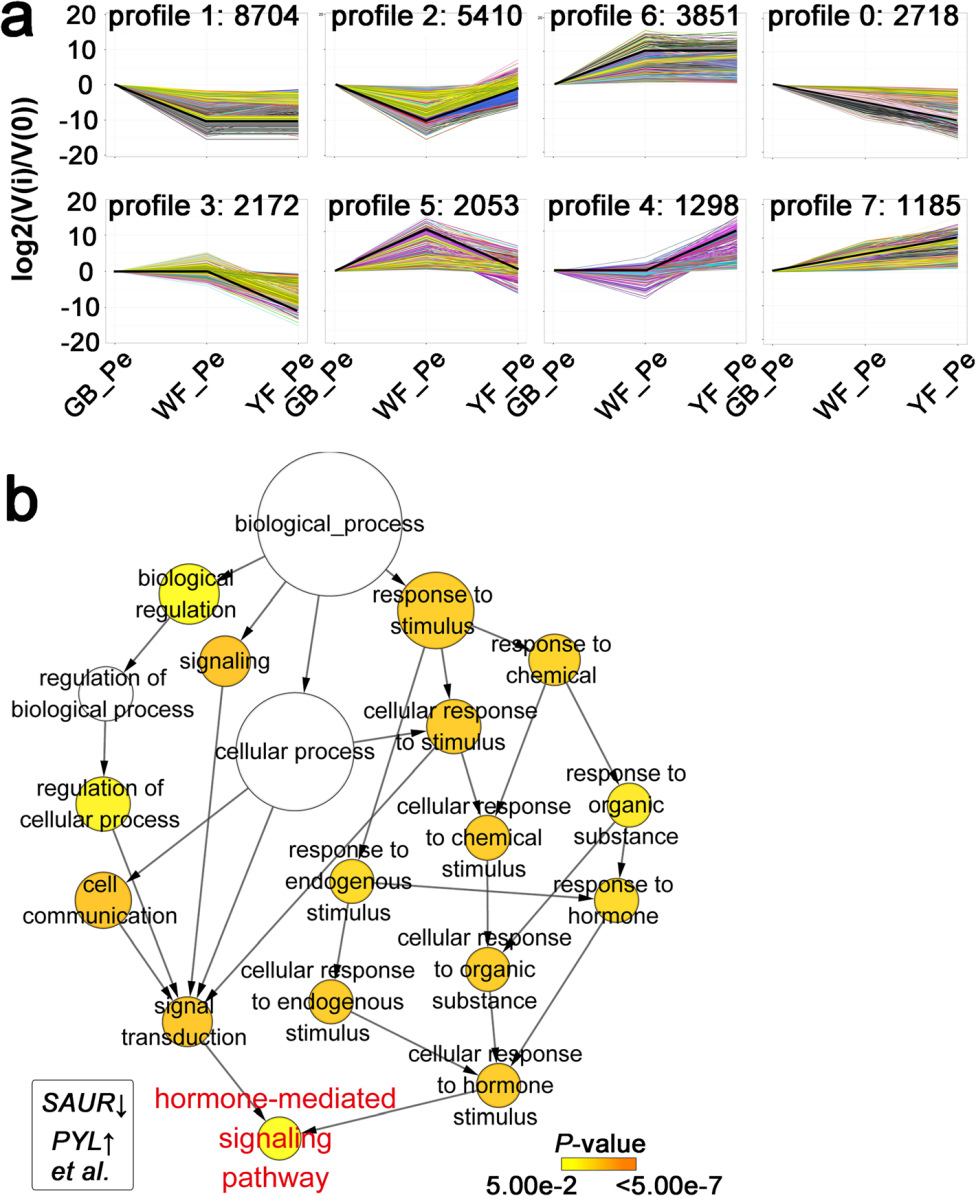
Analysis of DEGs. **a** Cluster analysis of all DEGs from the expression profiles. Black lines indicated the average expression level of unigenes grouped into the same profile. **b** Enrichment of selected GO terms for DEGs (> 1 RPKM) selected from profiles 3 and 4. The biological process with false discovery rate (adjusted ***P*** *<* 0.05, Student’s ***t-***test) is shown

All 29,679 identified DEGs were further classified into 8 clusters on the basis of expression alterations during petal colour transition (Fig. 3a). A total of 3470 DEGs were classified into two profiles based on expression changes across the three developmental stages: expression stable and then increased (profile 4) and expression stable and then decreased (profile 3). The opposite change patterns of gene expression during the petal colour transition from white to yellow suggest a tight linkage of these genes with petal colour transition in *L. japonica*.

GO enrichment analysis was further performed to investigate the biological functions of these 1897 DEGs (RPKM > 1 in at least one sample from the 3470 DEGs) that showed higher or lower expression in YF_Pe. The hormone-mediated signalling pathway was significantly enriched in the biological process subcategory (Fig. 3b). DEGs involved in hormone-mediated signalling pathways, such as *small auxin-up RNA* (*SAUR*) and *PYRABACTIN RESISTANCE1-like* (*PYL*), were significantly differentially expressed between yellow petals and non-yellow petals and seemed relevant to the goal of this study.

### Analysis of DEGs involved in hormone-mediated signalling pathways

GO enrichment analysis showed that DEGs were mainly enriched in hormone-mediated signalling pathways. To better investigate hormonal regulation in the colour transitions, we analysed the 67 DEGs (> 1 RPKM) that were enriched in the signalling pathways of auxin, cytokinin, gibberellin, BR, jasmonic acid, ABA and ethylene in YF_Pe vs GB_Pe and YF_Pe vs WF_Pe (Fig. S5 and Table S7).

In the auxin signalling pathway, 15 DEGs were identified, of which the *AUX1*, *TIR1*, *ARF* and *SAUR* genes were significantly downregulated from GB_Pe to YF_Pe, while three *IAA*s were upregulated at WF_Pe (Fig. S5a). A total of 18 DEGs were enriched in the cytokinine signalling pathway, including HKs, HPs, type-B RRs and type-A RRs. All of these DEGs were downregulated from GB_Pe to WF_Pe and YF_Pe (Fig. S5b). Meanwhile, in the gibberellin signalling pathway, *GID1*, *GID2* and *DELLA* genes were identified and significantly downregulated in YF_Pe (Fig. S5c). In the BR signalling pathway, 13 DEGs were identified, most of which were first downregulated and then upregulated in the transition. Specifically, the expression of *BRI1*, *BSK*, *BZR1_2*, *CYCD3* and *TCH4* was significantly higher in YF_Pe than in WF_Pe (Fig. S5d). Four DEGs were enriched in the jasmonic acid signalling pathway, and their expression levels were higher in GB_Pe than in WF_Pe and YF_Pe (Fig. S5e). Furthermore, *JAR1*, *COI-1* and *MYC2* were expressed at higher levels in YF_Pe than in WF_Pe, while *JAZ* was expressed at lower levels in YF_Pe than in WF_Pe. However, seven DEGs were identified in the ABA signalling pathway, including *PYL*, *PP2C*, *SNRK2* and *ABF*, of which *PYLs* and *SNRK2* were significantly upregulated in YF_Pe (Fig. S5f). In the ethylene signalling pathway, five DEGs were identified, of which *EIN3*, *ERS* and *ERF1* were significantly upregulated in YF_Pe (Fig. S5g).

### Analysis of pigment-related DEGs during petal colour transitions

**Fig. 4:**
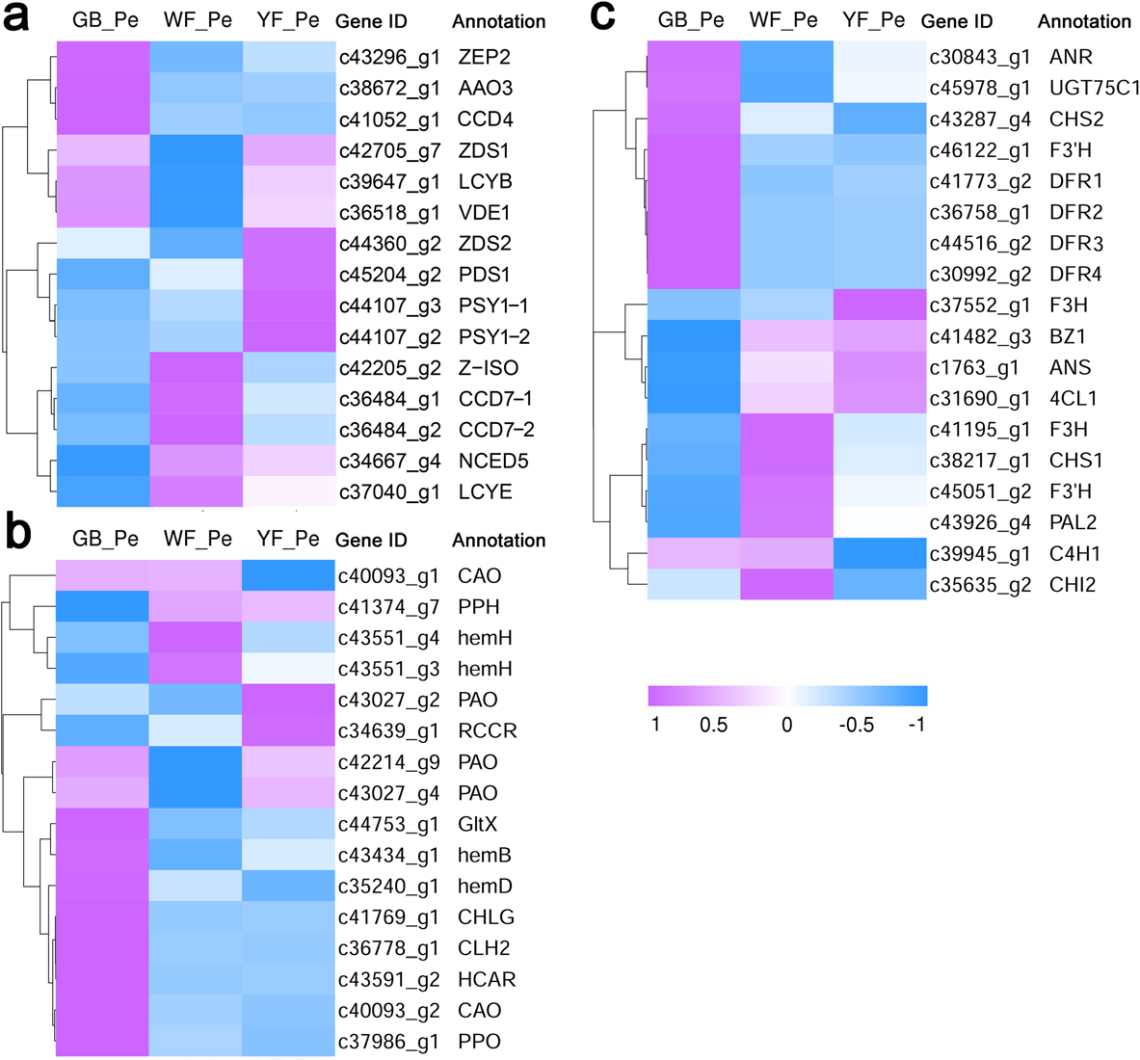
Expression levels of the pigments synthesis/catabolism in GB_Pe, WF_Pe and YF_Pe. **a** DEGs of carotenoid metabolism-related genes. **b** DEGs of porphyrin and chlorophyll metabolism-related genes. **c** DEGs of flavonoid/anthocyanin metabolism-related genes. High expression levels are represented in orchid. Low expression levels are represented in blue

To investigate the pathways of pigment synthesis/degradation during the transitions, the expression levels of carotenoid, anthocyanin and chlorophyll metabolism-related genes were analysed. A total of 49 DEGs (> 1 RPKM) regulating carotenoid, anthocyanin and chlorophyll metabolism were identified and significantly differentially expressed between yellow petals and non-yellow petals (GB_Pe or WF_Pe) (Fig. 4 and Table S7).

In the carotenoid biosynthesis pathway, *PSY1*, *PDS1*, *ZDS1* and *ZDS2* were significantly upregulated in YF_Pe. However, three carotenoid degradation-related genes, *carotenoid cleavage dioxygenase 4* (*CCD4*), *CCD7* and *abscisic-aldehyde oxidase 3* (*AAO3*), were significantly downregulated in YF_Pe (Fig. 4a). Meanwhile, the expression levels of chlorophyll metabolism-related genes showed significant differences. Among these genes, biosynthesis-related genes, including *glutamyl-tRNA synthetase* (*GltX*), *protoporphyrinogen IX oxidase* (*PPO*) and *chlorophyll synthase* (*CHLG*), were significantly upregulated in GB_Pe. However, *pheophytinase* (*PPH*), *pheophorbide a oxygenase* (*PAO*) and *red chlorophyll catabolite reductase* (*RCCR*) were significantly downregulated in GB_Pe (Fig. 4b).

In the basic upstream pathway of flavonoid/anthocyanin biosynthesis, some DEGs were identified in WF_Pe vs GB_Pe, YF_Pe vs WF_Pe and/or YF_Pe vs GB_Pe comparisons, such as *phenylalanine ammonia-lyase* (*PAL*), *trans-cinnamate 4-monooxygenase* (*C4H*), *4-coumarate-CoA ligase* (*4CL*), *chalcone synthase* (*CHS*), *chalcone isomerase* (*CHI*), *flavanone 3β-hydroxylase* (*F3H*), *flavonoid 3′-monooxygenase* (*F3’H*), *dihydroflavonol 4-reductase* (*DFR*) and *ANS*. In the specific anthocyanin downstream branch, one *anthocyanidin 3-O-glucosyltransferase* (*BZ1*) gene and one *anthocyanidin 3-O-glucoside 5-O-glucosyltransferase* (*UGT75C1*) gene were identified as DEGs. The expression levels of *ANS* and *BZ1* were significantly upregulated in YF_Pe. In contrast, some DEGs, including *CHS2*, *DFR*s and *UGT75C1*, were upregulated in GB_Pe. Although the expression levels of four *DFR*s and one *UGT75C1* first declined in white petals, they then slightly rose from WF_Pe to YF_Pe (Fig. 4c).

### Validation of the expression analysis of key genes

**Fig. 5:**
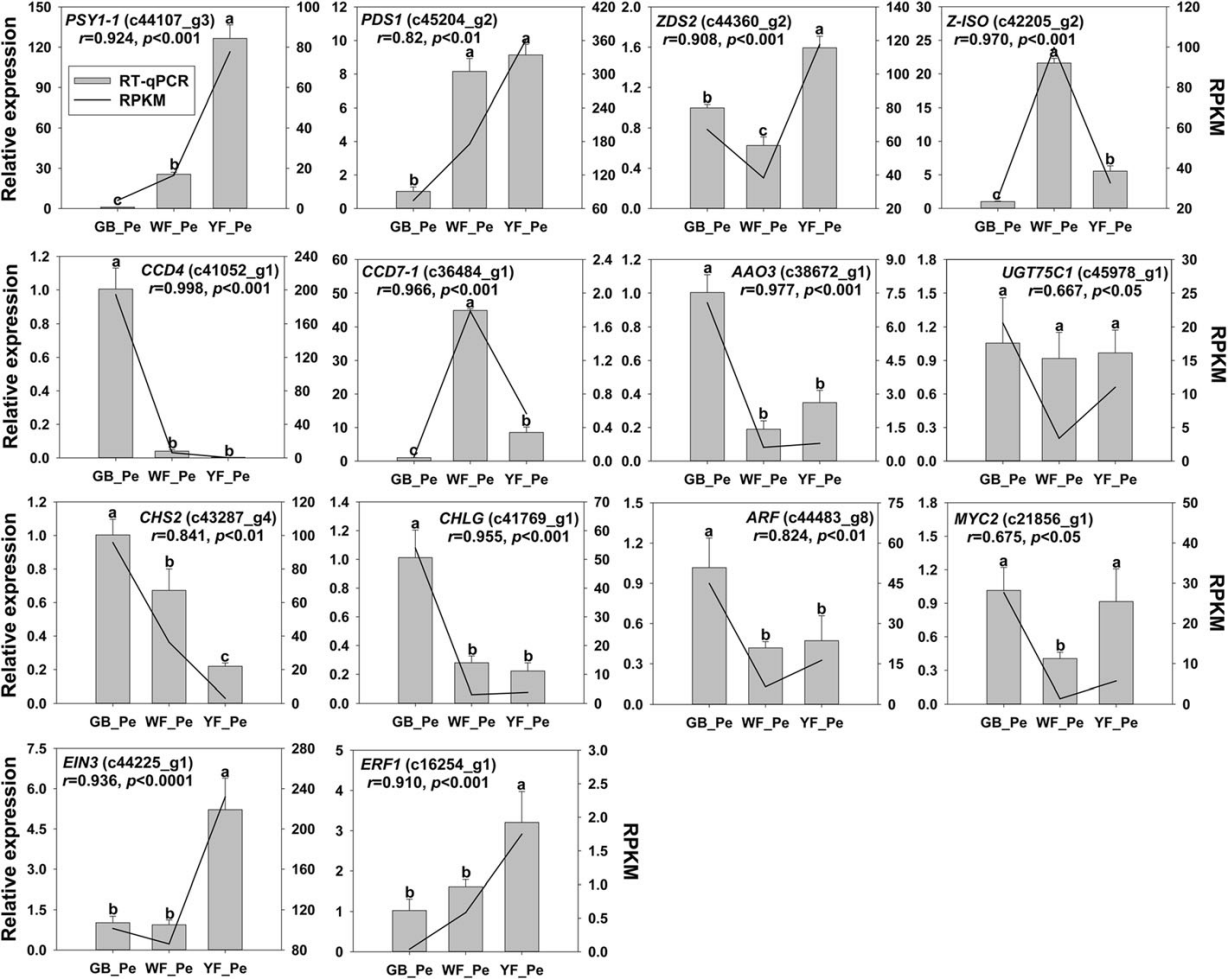
Validation of the expression of pigment-related genes in *L. japonica* by RT-qPCR. Error bars indicate the standard deviation of three independent biological repeats. Significant differences are indicated by different letters at *P* < 0.05

A total of fourteen unigenes in pigment metabolism pathways or hormone-mediated signalling pathways were randomly selected and identified by RT-qPCR. The expression patterns of these DEGs corresponded well with the RPKM values obtained by RNAseq (Fig. 5). Pearson correlation analysis showed high correlation coefficients between the RNA-seq and RT-qPCR data, suggesting that the sequencing data are reliable.

## Discussion

### Pigment accumulation and metabolism-related genes

During petal colour transitions, a previous study reported that the concentrations of total chlorophyll significantly decreased from green buds to white flowers and then remained stable to yellow flowers (22). In this study, the expression levels of chlorophyll biosynthesis-related genes, including *GltX*, *PPO* and *CHLG*, were significantly downregulated in WF_Pe and YF_Pe compared with GB_Pe (Fig. 4b). As known to us, *CHLG* is involved in the final step in chlorophyll synthesis [38], the level of *CHLG* expression showed a significant correlation with chlorophyll content in the petals of *L. japonica*. In addition to chlorophyll biosynthesis-related genes described above, the expression levels of *chlorophyllide a oxygenase* (*CAO)* and *hydroxymethyl chlorophyll a reductase* (*HCAR)*, which regulate the interconversion of chlorophyll a and chlorophyll b and play a crucial role in greening processes [39–41], also drastically decreased in WF_Pe and YF_Pe. In a previous study, the expression level of *HCAR* showed a positive correlation with chlorophyll content in pale-green petals of carnation [42]. In contrast, chlorophyll degradation-related genes, such as *PPH*, *PAO* and *RCCR*, were significantly upregulated in YF_Pe. These results revealed that the decrease in chlorophyll accumulation mainly accounted for the loss of visual green colour in WF_Pe and YF_Pe.

The total contents of chlorophyll and carotenoid were both found to decrease to their lowest levels in WF_Pe (22). Accordingly, the expression of chlorophyll and carotenoid biosynthesis genes was significantly downregulated in WF_Pe. Interestingly, some anthocyanins were detected in WF_Pe, such as delphinidin and petunidin 3-O-glucoside, suggesting the presence of unobservable pigments in white petals.

In flowering plants, carotenoids mainly participate in petal colours ranging from yellow to red [3]. Several plant lineages have yellow flowers and contain pigments derived from carotenoids [5]. A previous study reported that the content of total carotenoids dramatically increased from the WF to YF stages [22, 24]. Similarly, most of the detected carotenoids were significantly upregulated in YF_Pe compared with WF_Pe in this study (Table 1). Among the 10 significantly higher carotenoids recorded in YF_Pe, the top four carotenoids were α-carotene, zeaxanthin, violaxanthin and γ-carotene. These four carotenoids may be the major contributors to yellow color of YF_Pe.

In accordance with the changes in carotenoid content, carotenoid biosynthesis-related genes, such as *PSY1*, *PDS1*, *ZDS1*, *ZDS2*, *LCYB* and *VDE*, were significantly upregulated in YF_Pe compared with WF_Pe (Fig. 4a), and similar results were reported in a previous study [24]. Because of their key roles in regulating carotenoid biosynthesis, *PSY*, *PDS* and *ZDS* have been subjected to intensive investigation. Previously, in the steps of carotenoid biosynthesis, *PSY* was shown to be involved in the condensation of two geranylgeranyl diphosphate molecules into phytoene, and the upregulation of *PSY* enhances carotenoid accumulation [43–48]. Then, the phytoene is subjected to a series of desaturation reactions catalysed by carotene desaturases, such as PDS and ZDS [4, 44]. Similar observations of petal colours have been reported in monocots, such as *Lilium* and *Oncidium* [7, 49]. In different cultivars of Asiatic hybrid lily, the petal colours are correlated well with the transcription levels of biosynthetic genes, including *PSY*, *PDS* and *ZDS* [49].

In contrast, the expression levels of the carotenoid catabolism-related genes *CCD4* and *CCD7* were significantly downregulated in YF_Pe. It has been found that the expression of *CCD4* affects carotenoid levels in various plants. As reported for pigments of *Brassica* and *Dendranthema*, the increase in carotenoid content is related to disruption of a *CCD4* gene involving the petal colour transition from white to yellow [50, 51]. In rose cultivars of yellow petals, carotenoid degradation has a high correlation with the expression of *RhCCD4* [52].

Furthermore, we found that two anthocyanins (pelargonidin and cyanidin) were significantly upregulated in YF_Pe. In particular, the pelargonidin and cyanidin concentrations both tripled from WF_Pe to YF_Pe. In *Gentiana lutea* L. var. *aurantiaca*, the orange petal colour is predominantly caused by newly synthesized pelargonidin glycosides, which confer a reddish hue to the yellow background colour derived from carotenoids [53]. Pelargonidin and cyanidin accumulated at increasing concentrations from WF_Pe to YF_Pe, in sync with the variation in carotenoid content, which might assist the petal colour transition from a white to golden appearance.

Herein, the expression levels of some key anthocyanin biosynthesis-related genes, such as *DFR*s, *ANS*, *BZ1* and *UGT75C1*, were upregulated in YF_Pe compared with WF_Pe (Fig. 4c). As an important step in the anthocyanin biosynthetic pathway, *DFR* is associated with anthocyanin colouration in flowers, such as *Freesia hybrida* [54], Andean genus *Iochroma* (Solanaceae) [55] and Cape *Erica* species [56]. In the present study, four *DFR* genes were identified and increased from WF_Pe to YF_Pe. To our knowledge, ANS is considered the first key enzyme that could lead to flavonoid flux into the anthocyanin branch. The expression level of *ANS* was upregulated in YF_Pe, which accounted for the increased concentrations of pelargonidin and cyanidin.

### Changes in hormones concentrations associated with pigment accumulation

Plant hormones are involved in all stages of flower development. In our study, changes in the concentrations of IAA, ZR, GA_3_, BR, MeJA and ABA revealed that the transitions are regulated by endogenous hormones. Accordingly, the key DEGs involved in the hormone signal transduction pathways were significantly enriched in the auxin, cytokinin, gibberellin, BR, jasmonic acid, ABA and ethylene signalling pathways.

In this study, the concentration of IAA was significantly decreased from GB_Pe to YF_Pe (Fig. 2a). Accordingly, the genes from the auxin signalling pathway were mostly downregulated in YF_Pe (Fig. S5a). For example, *SAUR* genes, a family of auxin-responsive genes in auxin signalling pathways, were downregulated in the transitions. However, compared with WF_Pe, the expression levels of three *IAA*s were significantly downregulated in YF_Pe. It was previously reported that IAA represses the transcription of carotenoid biosynthesis-related genes, e.g., *PSY*, *ZISO*, *PDS* and *CRTISO* [57]. This indicated that downregulation of the *IAA* genes play important roles in carotenoid accumulation.

The concentrations of BR and MeJA were both slightly higher in YF_Pe than in WF_Pe (Fig. 2d, e). The expression levels of most genes from the BR and jasmonic acid signalling pathways, including *BRI1*, *BSK*, *BZR1_2*, *TCH4*, *CYCD3*, *JAR1*, *COI-1* and *MYC2*, were upregulated in YF_Pe (Fig. S5d, e). In *Solanum lycopersicum*, the application of BR was previously reported to increase carotenoid accumulation [58]. Furthermore, ectopic expression of *BZR1-1D* results in an increase in carotenoid accumulation [59]. Meanwhile, the exogenous application of MeJA in the *Never ripe* mutant of *Solanum lycopersicum* significantly enhanced lycopene accumulation, as well as the expression levels of *PSY1* and *PDS* [60]. In the present study, the expression levels of *PSY1* and *PDS1* were significantly higher in YF_Pe than in WF_Pe (Fig. 4a).

Genes from the ethylene and ABA signalling pathways were significantly upregulated during petal colour transitions (Fig. S5f, g). Previous studies indicate that upregulation of ethylene-related genes accelerates chlorophyll degradation [61] and carotenoid accumulation [57]. In our study, the upregulation of *EIN3*, *ERS* and *ERF1* might lead to chlorophyll degradation and carotenoid accumulation in YF_Pe. In the ABA signalling pathway, the expression level of *ABF* reached the highest level in WF_Pe; *PYL* and *SNRK2* were upregulated in WF_Pe and YF_Pe. In Arabidopsis, overexpression of *ABF* is associated with triggering chlorophyll degradation [62–64]. ABA signalling pathway related genes upregulated in WF_Pe and YF_Pe along with the increased concentration of ABA during flower development play roles in petal color transitions.

## Conclusions

**Fig. 6:**
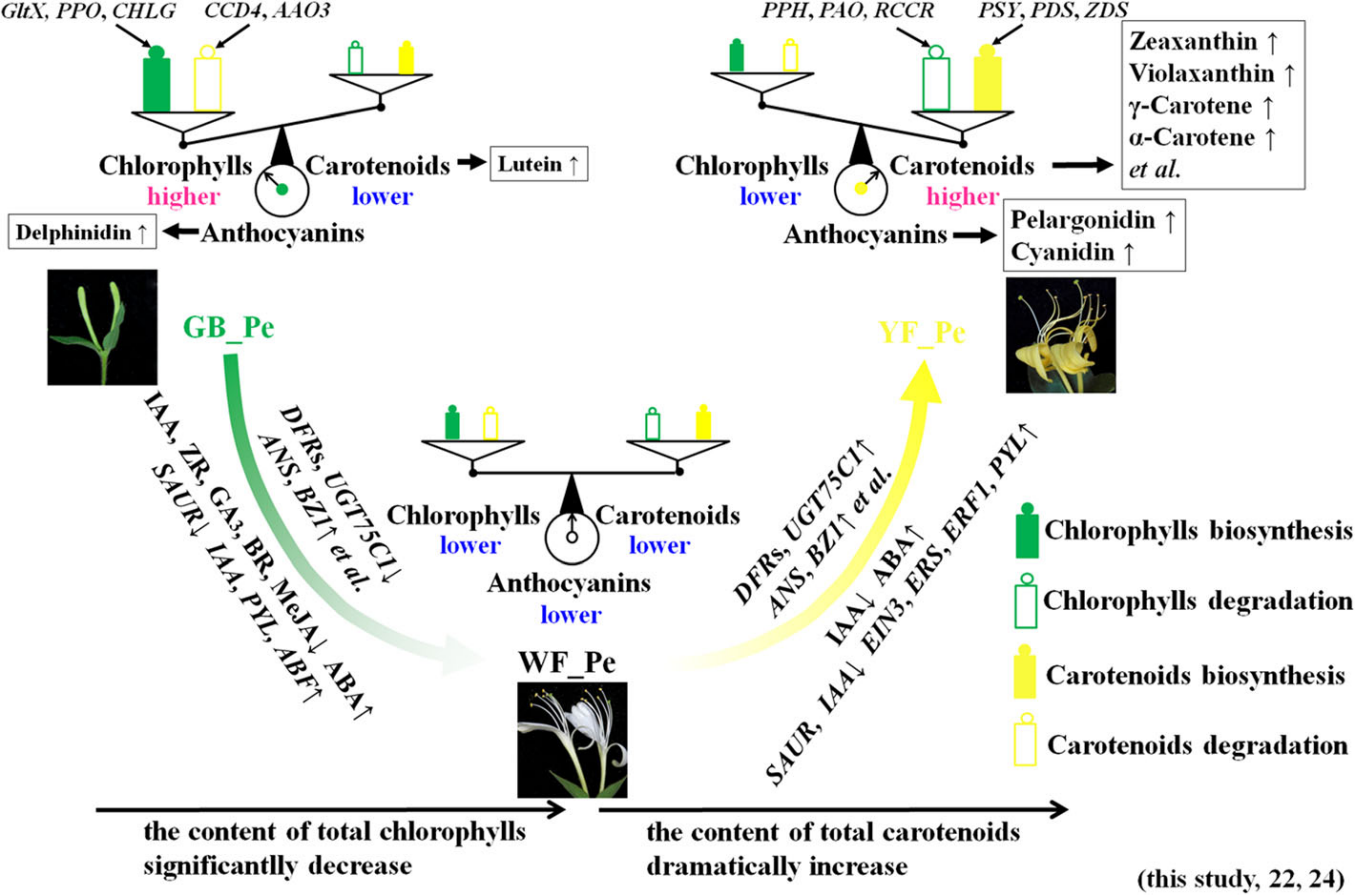
Schematic of changes in the regulatory genes and metabolites in petal color- transition in *L. japonica*

In this study, the most comprehensive metabolome, hormone, and transcriptome analyses investigated petal colour transitions in *L. japonica*. Analyses of key candidate genes, metabolites and hormones highlighted the effects of carotenoids, anthocyanins and endogenous hormones; this enabled us to clarify the regulatory mechanisms underlying the transitions. Based on our results and previously published studies, we provide a conceptual model for the regulatory network of the transitions in *L. japonica* (Fig. 6). In this model, the existing chlorophyll/carotenoid balance is disturbed during flower development. Overall, the content of total chlorophylls decreased in WF_Pe and YF_Pe, along with low expression of chlorophyll synthesis genes and high expression of chlorophyll degradation genes, resulting in green colour loss with flower development. In contrast, although the content of total carotenoids first dropped in WF_Pe, it then rapidly accumulated from WF_Pe to YF_Pe, in sync with the increase in the expression of genes related to carotenoid biosynthesis. The ten highest concentrations carotenoids were detected in YF_Pe, and the top four carotenoids were α-carotene, zeaxanthin, violaxanthin and γ-carotene, which are major contributors to yellow color of YF_Pe. It was also found that a few anthocyanins differentially accumulated during flower development, indicating that they may assist with to vivid colour of GB_Pe and YF_Pe. Meanwhile, this developmental process is regulated by endogenous hormones. These variations in key pigment and hormone-mediated signalling pathway-related genes, pigments and hormones promote petal colour transitions from green to white and then to yellow in *L. japonica*.

## Supplementary Information

**Additional file 1: Figure S1.** Length distribution of *L. japonica* transcriptome.

**Additional file 2: Figure S2.** GO functional analysis and classification of *L. japonica* transcriptome.

**Additional file 3: Figure S3.** KEGG classification of *L. japonica* transcriptome.

**Additional file 4: Figure S4.** The numbers of DEGs among three *L. japonica* petals**. a** The relationships among the three DEGs datasets. **b** The number of DEGs in each comparison.

**Additional file 5: Figure S5.** Expression profiles of DEGs that regulate plant hormone signal transduction in GB_Pe, WF_Pe, and YF_Pe. Orchid, high expression levels; blue, low expression levels. Genes encoding key enzymes of auxin (**a**), cytokinine (**b**), gibberellin (**c**), brassinosteroid (**d**), jasmonic acid (**e**), abscisic acid (**f**) and ethylene (**g**) signaling pathways were exhibited.

**Additional file 6: Table S1.** Primers used for reverse transcription quantitative PCR (RT-qPCR). **Table S2.** Color parameters in GB_Pe, WF_Pe, and YF_Pe**. Table S3.** Differentially-accumulated anthocyanins in WF_Pe vs GB_Pe, YF_Pe vs GB_Pe and/or YF_Pe vs WF_Pe**. Table S4.** Throughput and quality of *L. japonica* transcriptome data. **Table S5.** Summary of *L. japonica* de novo transcriptome assembly. **Table S6.** Mapping results of *L. japonica* unigenes to various databases.

**Additional file 7: Table S7.** Genes involving in plant hormone signal transduction and pigments metabolism.

## Data Availability

These sequence data have been submitted to the SRA database under accession number PRJNA574570. The datasets supporting the conclusions of this article are included within the article and its additional files.

## Abbreviations

GB_Pe: Green bud petal
WF_Pe: White flower petal
YF_Pe: Yellow flower petal
DEGs: Differentially expressed genes
GA_3_: Gibberellic acid
BR: Brassinosteroid
ABA: Abscisic acid
IAA: Indoleacetic acid
ZR: Zeatin riboside
MeJA: Methyl jasmonate
MS: Mass spectrometry
QC: Quality control
ACN: Acetonitrile
ANOVA: Analysis of variance
GO: Gene ontology
KEGG: Kyoto Encyclopedia of Genes and Genomes
RPKM: Reads per kilobase millon mapped reads
RT-qPCR: Reverse transcription quantitative PCR
ANS: Anthocyanidin synthase
PSY: Phytoene synthase
PDS: Phytoene desaturase
ZDS: ζ-Carotene desaturase
SAUR: Small auxin-up RNA
PYL: PYRABACTIN RESISTANCE1-like
CCD: Carotenoid cleavage dioxygenase
AAO3: Abscisic-aldehyde oxidase 3
GltX: Glutamyl-tRNA synthetase
PPO: Protoporphyrinogen IX oxidase
CHLG: Chlorophyll synthase
PPH: Pheophytinase
PAO: Pheophorbide a oxygenase
RCCR: Red chlorophyll catabolite reductase
CAO: Chlorophyllide a oxygenase
HCAR: Hydroxymethyl chlorophyll a reductase
PAL: Phenylalanine ammonia-lyase
C4H: Trans-cinnamate 4-monooxygenase
4CL: 4-Coumarate-CoA ligase
CHS: Chalcone synthase
CHI: Chalcone isomerase
F3H: Flavanone 3β-hydroxylase
F3’H: Flavonoid 3′-monooxygenase
DFR: Dihydroflavonol 4-reductase
BZ1: Anthocyanidin 3-O-glucosyltransferase
UGT75C1: Anthocyanidin 3-O-glucoside 5-O-glucosyltransferase
