# Supplementary figures and images for: Integrated metabolic profiling and transcriptome analysis of pigment accumulation in Lonicera japonica flower petals during colour-transition
Source: BMC Plant Biol. 2021 Feb 17;21:98. doi: 10.1186/s12870-021-02877-y (PMC7890969; doi:10.1186/s12870-021-02877-y)

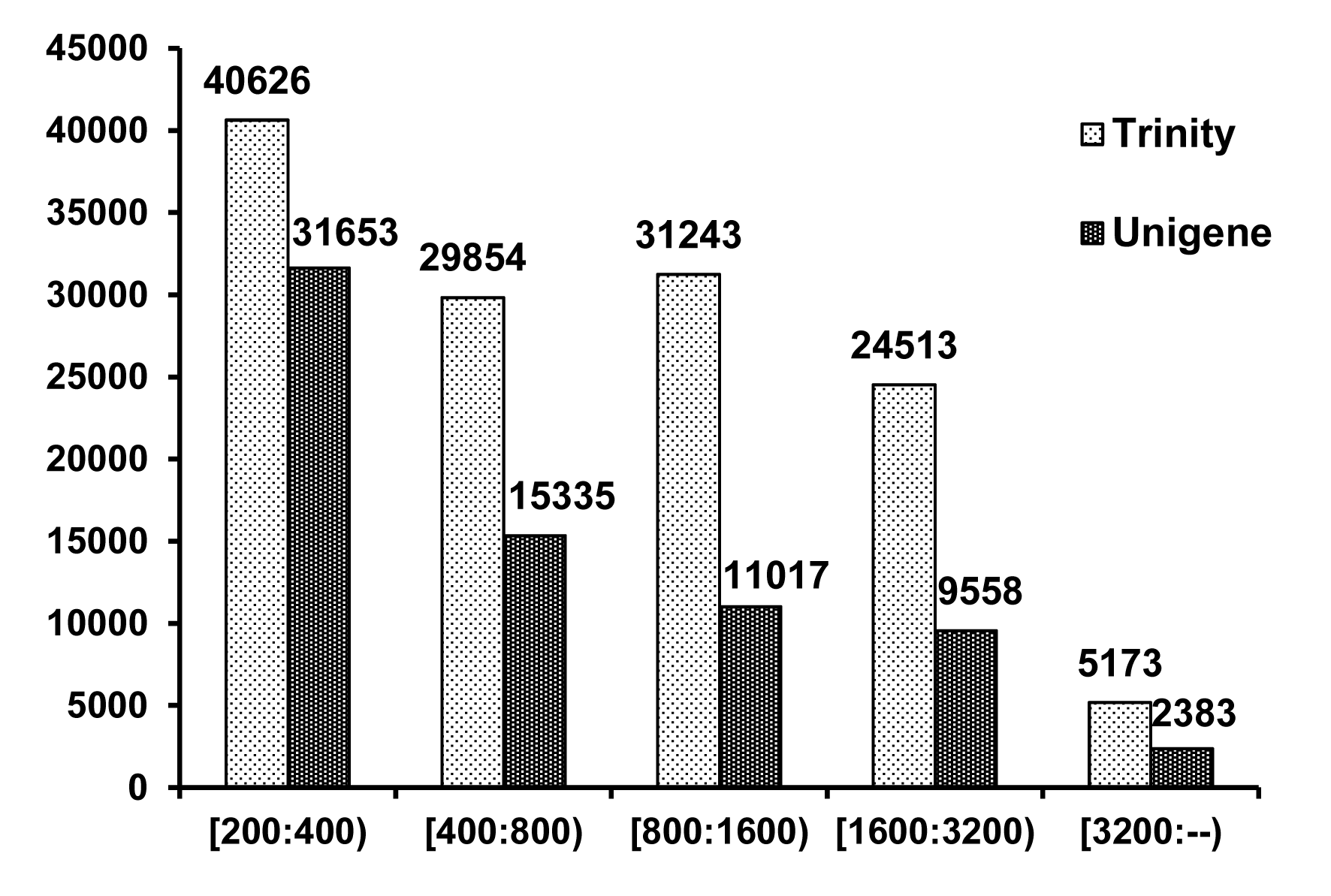

Supplement: Supplementary file 1 — Additional file 1: Figure S1. Length distribution of L. japonica transcriptome. [file 12870_2021_2877_MOESM1_ESM.tif]

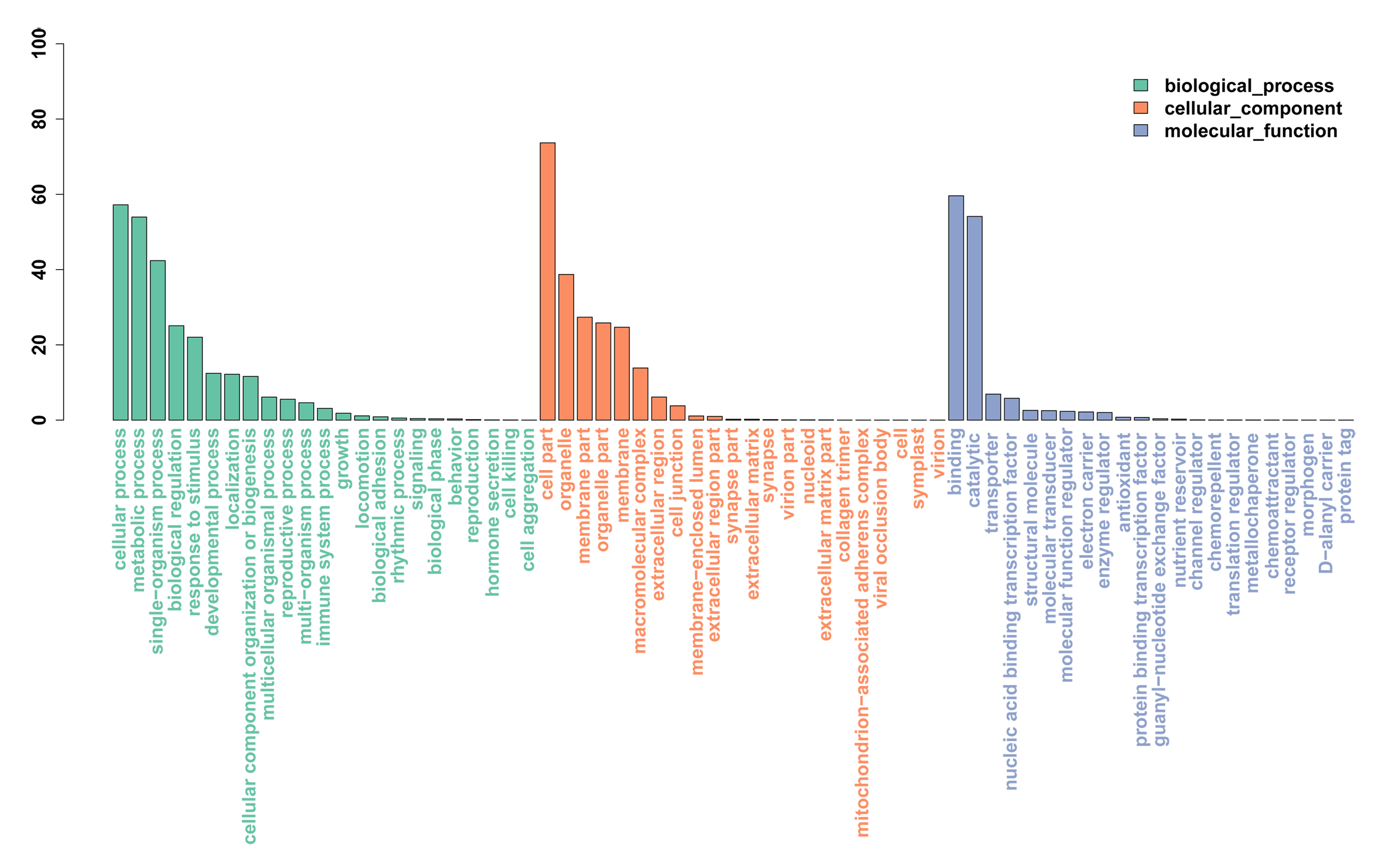

Supplement: Supplementary file 2 — Additional file 2: Figure S2. GO functional analysis and classification of L. japonica transcriptome. [file 12870_2021_2877_MOESM2_ESM.tif]

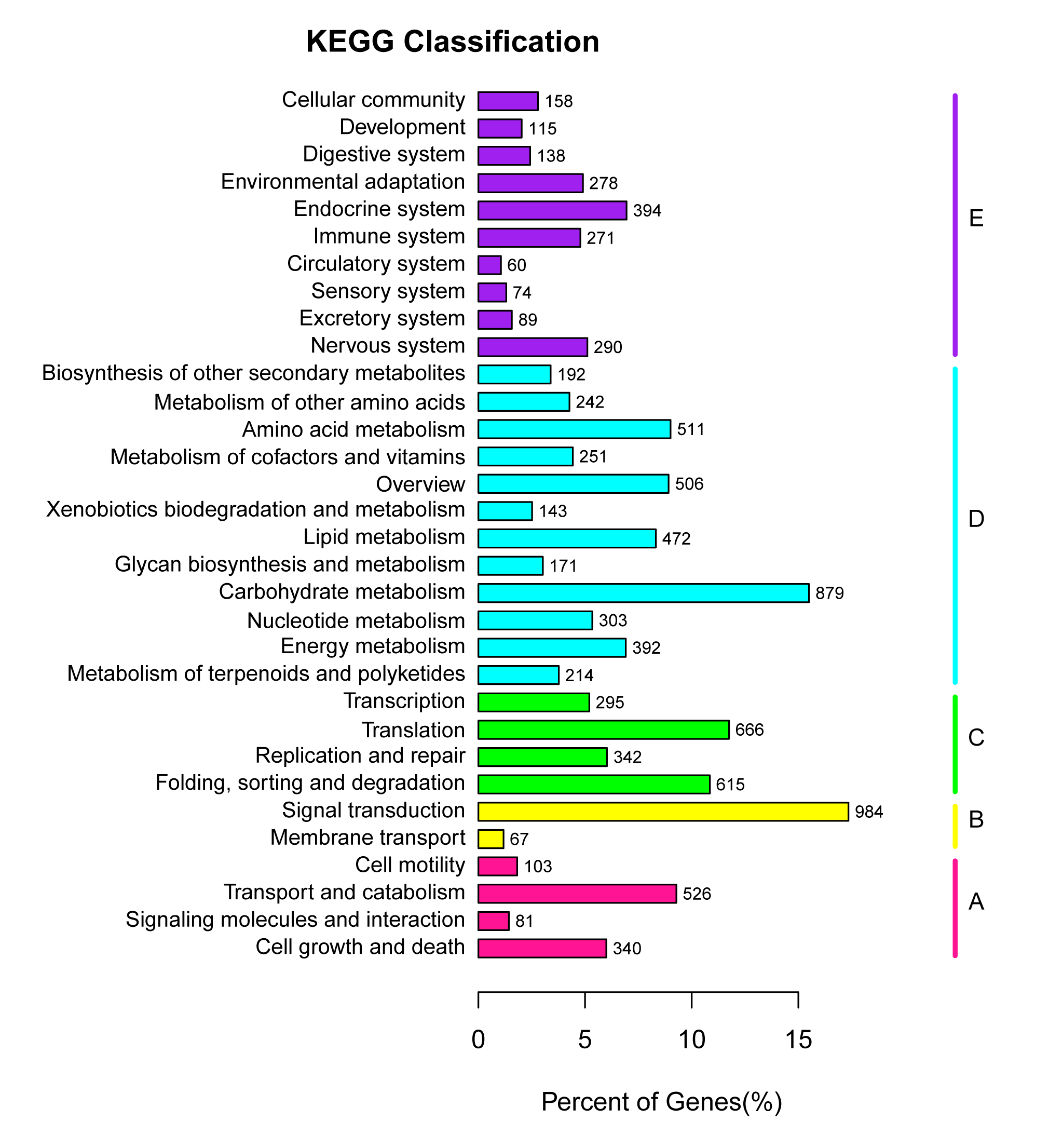

Supplement: Supplementary file 3 — Additional file 3: Figure S3. KEGG classification of L. japonica transcriptome. [file 12870_2021_2877_MOESM3_ESM.tif]

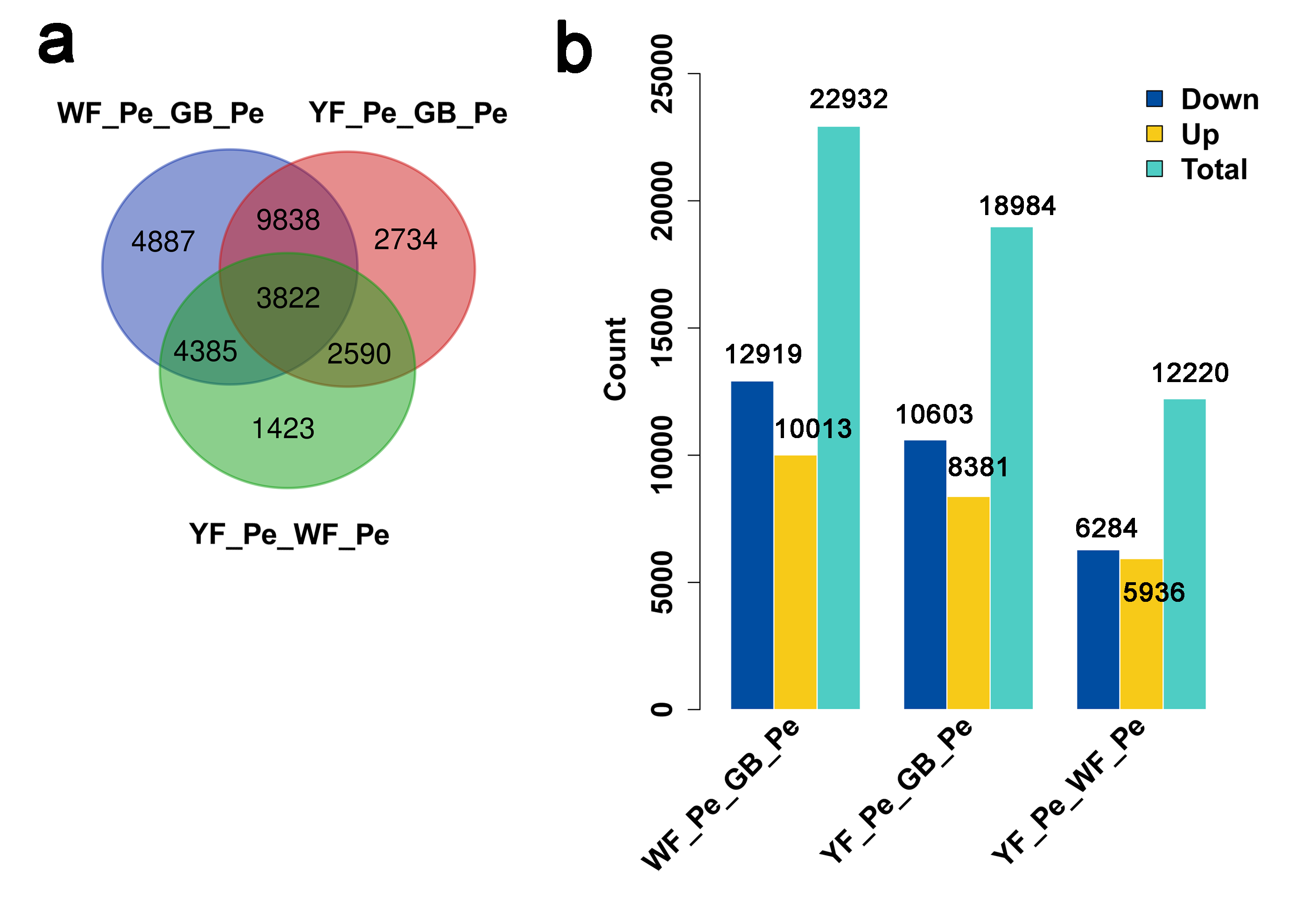

Supplement: Supplementary file 4 — Additional file 4: Figure S4. The numbers of DEGs among three L. japonica petals. a The relationships among the three DEGs datasets. b The number of DEGs in each comparison. [file 12870_2021_2877_MOESM4_ESM.tif]

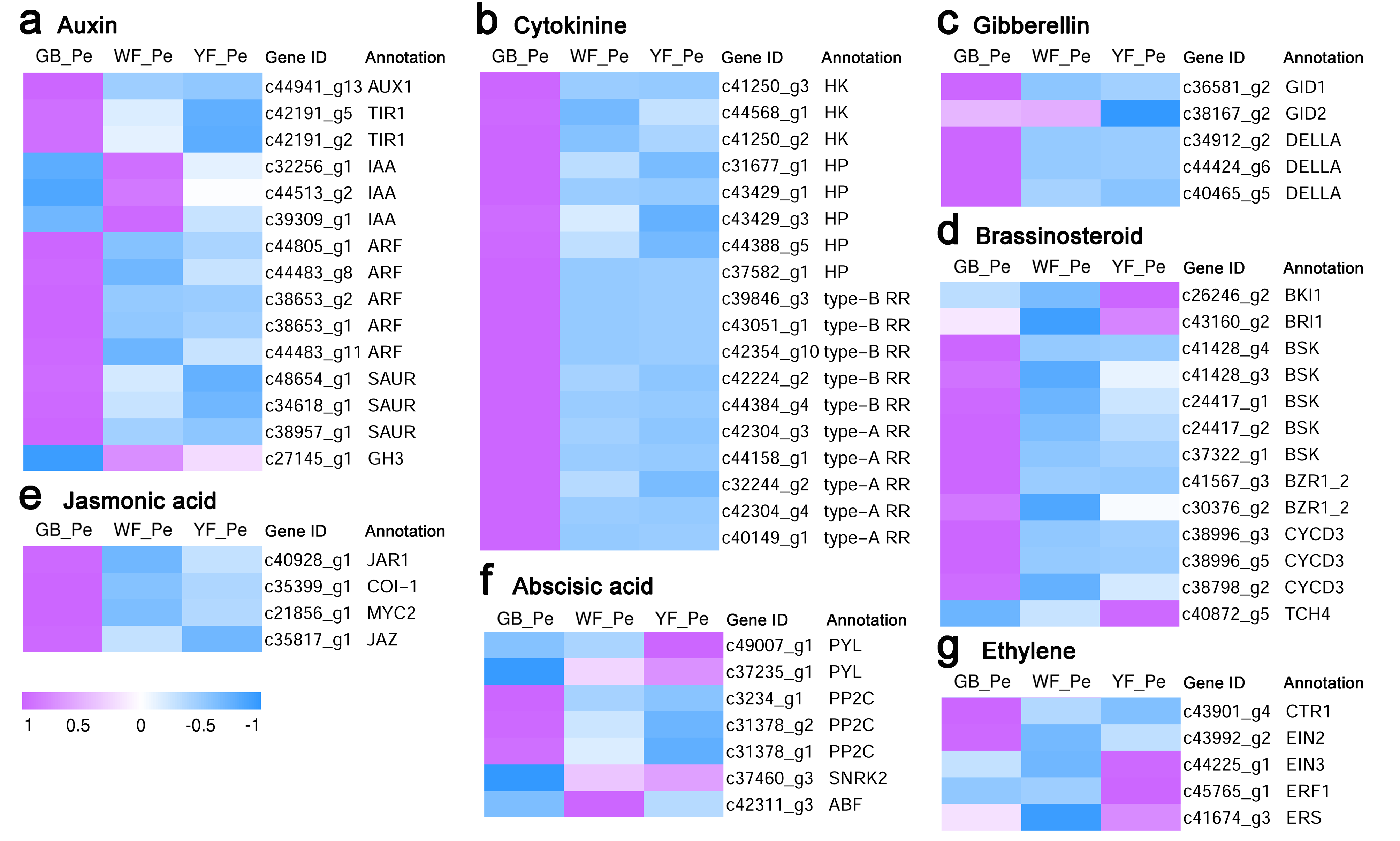

Supplement: Supplementary file 5 — Additional file 5: Figure S5. Expression profiles of DEGs that regulate plant hormone signal transduction in GB_Pe, WF_Pe, and YF_Pe. Orchid, high expression levels; blue, low expression levels. Genes encoding key enzymes of auxin (a), cytokinine (b), gibberellin (c), brassinosteroid (d), jasmonic acid (e), abscisic acid (f) and ethylene (g) signaling pathways were exhibited. [file 12870_2021_2877_MOESM5_ESM.tif]
