# Supplementary material for: Integrated metabolic profiling and transcriptome analysis of pigment accumulation in Lonicera japonica flower petals during colour-transition
Source: BMC Plant Biol. 2021 Feb 17;21:98. doi: 10.1186/s12870-021-02877-y (PMC7890969; doi:10.1186/s12870-021-02877-y)
Supplement: Supplementary file 6 — Additional file 6: Table S1. Primers used for reverse transcription quantitative PCR (RT-qPCR). Table S2. Color parameters in GB_Pe, WF_Pe, and YF_Pe. Table S3. Differentially-accumulated anthocyanins in WF_Pe vs GB_Pe, YF_Pe vs GB_Pe and/or YF_Pe vs WF_Pe. Table S4. Throughput and quality of L. japonica transcriptome data. Table S5. Summary of L. japonica de novo transcriptome assembly. Table S6. Mapping results of L. japonica unigenes to various databases. [file 12870_2021_2877_MOESM6_ESM.docx]

**Supplementary materials**

**Table S1** Primers used for reverse transcription quantitative PCR (RT-qPCR)

| Gene | Forward primer (5’ to 3’) | Reverse primer (5’ to 3’) | Product size (bp) |
| --- | --- | --- | --- |
| *PSY1-1* (c44107_g3) | CTCGATGCTGCTTTATCCG | TGTGATATAGGTGCAATTCCC | 200 |
| *PDS 1*(c45204_g2) | GTATTAATGACCGATTGCAG | TTCAACATAGGCTTGTCCA | 221 |
| *ZDS2* (c44360_g2) | ATTTATATGCTTGAGTTATGC | CATTGTGCATCTAGGGTCTGT | 203 |
| *Z-ISO* (c42205_g2) | AATTCGATTTCGCCAAGCAGA | CTAGCCAAGCCACTATGGAC | 214 |
| *CCD4* (c41052_g1) | AATCCAGCTTTCGTCGCTA | CATCGTCCTCCTTCGCATT | 220 |
| *CCD7-1* (c36484_g1) | AAGTTATTGCCTCGCCTTGTT | TCACCACTGTATCAAACGGAA | 217 |
| *AAO3* (c38672_g1) | AAGATTTAGGAGCACCCAT | CTTCATCTGGAACCGCAAG | 213 |
| *UGT75C1* (c45978_g1) | AACCTAGAGCAAGATCCGAAC | AACCATCTTGTTTCCGGTCCA | 232 |
| *CHS2* (c43287_g4) | AGGTGGAGATTAAGCTGGGAC | CACCACAGTCTCAACAGTCAG | 215 |
| *CHLG* (c41769_g1) | CCGACATCTAACTGCATCCAT | GCTTTGTAAGCTGAACACGAA | 218 |
| *ARF* (c44483_g8) | TTGCACCTGTTCTTAATCGAG | CTTACTGAAGGAACACCGGAT | 220 |
| *MYC2* (c21856_g1) | TTATGATTCAACAAGCGACT | CACTAACATAGAACAACGACCAC | 218 |
| *EIN3* (c44225_g1) | TCATTAGGATTATTGGCTTCG | ATTCAAGGGAACAAGAACGC | 215 |
| *ERF1* (c16254_g1) | GAATCCTCTCTCTCGTGGGAC | CTCCGCTCTAGAATTCACCT | 231 |
| *Actin* (KY114518.1) | GCAAGTTATTACTATCGGAGCA | CCACTAAGCACAATGTTACCA | 168 |

**Table S2** Color parameters in GB_Pe, WF_Pe, and YF_Pe

| Group | parameters | Sample 1 | | | Sample 2 | | | Sample 3 | | | Sample 4 | | | Sample 5 | | | Mean | SD |
| --- | --- | --- | --- | --- | --- | --- | --- | --- | --- | --- | --- | --- | --- | --- | --- | --- | --- | --- |
| GB_Pe | L | 51.82 | 50.15 | 51.74 | 52.55 | 52.98 | 52.69 | 52.68 | 53.37 | 53.36 | 52.08 | 51.92 | 51.40 | 52.10 | 52.37 | 52.00 | 52.21 | 0.81 |
|  | a | -12.22 | -12.46 | -12.41 | -12.27 | -12.13 | -12.20 | -12.14 | -12.14 | -12.22 | -13.20 | -13.23 | -13.07 | -11.94 | -11.87 | -11.95 | -12.36 | 0.45 |
|  | b | 28.75 | 27.78 | 28.33 | 28.43 | 28.84 | 28.71 | 28.31 | 28.89 | 28.98 | 29.73 | 29.81 | 28.73 | 27.27 | 27.20 | 26.95 | 28.45 | 0.85 |
|  | c | 31.24 | 30.45 | 30.93 | 30.96 | 31.28 | 31.20 | 30.81 | 31.34 | 31.45 | 32.53 | 32.61 | 31.56 | 29.77 | 29.68 | 29.48 | 31.02 | 0.91 |
|  | h | 113.02 | 114.15 | 113.66 | 113.35 | 112.81 | 113.03 | 113.22 | 112.78 | 112.87 | 113.94 | 113.93 | 114.47 | 113.65 | 113.58 | 113.92 | 113.49 | 0.53 |
| WF_Pe | L | 80.87 | 81.18 | 79.53 | 84.60 | 80.42 | 81.96 | 85.30 | 81.60 | 83.66 | 82.44 | 84.25 | 81.57 | 83.21 | 84.05 | 81.40 | 82.40 | 1.69 |
|  | a | -0.40 | -0.26 | -0.99 | -0.58 | -0.30 | -0.43 | -0.98 | -0.40 | -0.35 | -0.64 | -0.62 | -1.02 | -0.54 | -0.42 | -0.72 | -0.58 | 0.25 |
|  | b | 13.26 | 13.60 | 13.32 | 13.47 | 12.83 | 13.49 | 17.19 | 14.99 | 16.45 | 14.62 | 17.07 | 13.35 | 14.76 | 14.38 | 15.24 | 14.53 | 1.43 |
|  | c | 13.26 | 13.61 | 13.36 | 13.51 | 12.83 | 13.50 | 17.31 | 15.00 | 16.50 | 14.64 | 17.13 | 13.35 | 14.90 | 14.52 | 15.30 | 14.58 | 1.45 |
|  | h | 88.28 | 88.90 | 85.75 | 94.32 | 88.64 | 91.85 | 96.57 | 92.86 | 94.68 | 92.51 | 94.69 | 89.64 | 94.33 | 91.20 | 89.70 | 91.59 | 3.05 |
| YF_Pe | L | 78.78 | 79.47 | 79.69 | 77.59 | 76.77 | 75.32 | 77.54 | 77.60 | 77.39 | 73.60 | 77.75 | 77.03 | 77.34 | 77.20 | 76.59 | 77.31 | 1.50 |
|  | a | 0.35 | 0.62 | 0.63 | 1.49 | 1.76 | 1.92 | 0.86 | 0.63 | 0.89 | 2.64 | 2.41 | 2.18 | 0.86 | 0.63 | 0.89 | 1.25 | 0.75 |
|  | b | 48.04 | 48.24 | 47.26 | 43.78 | 41.34 | 40.39 | 38.14 | 39.15 | 36.95 | 40.61 | 42.40 | 44.68 | 42.51 | 44.13 | 40.70 | 42.55 | 3.47 |
|  | c | 48.05 | 48.24 | 47.26 | 43.80 | 41.38 | 40.44 | 38.15 | 39.15 | 36.96 | 40.70 | 42.47 | 44.73 | 42.53 | 44.17 | 40.72 | 42.58 | 3.47 |
|  | h | 89.59 | 89.26 | 89.24 | 91.95 | 92.43 | 92.72 | 88.70 | 89.07 | 88.62 | 86.28 | 86.75 | 87.21 | 89.70 | 90.05 | 88.90 | 89.36 | 1.90 |

**Table S3.** Differentially-accumulated anthocyanins in WF_Pe vs GB_Pe, YF_Pe vs GB_Pe and/or YF_Pe vs WF_Pe

| Compounds | WF_Pe *vs* GB_Pe | | YF_Pe *vs* GB_Pe | | YF_Pe *vs* WF_Pe | |
| --- | --- | --- | --- | --- | --- | --- |
|  | VIP | Fold_Change | VIP | Fold_Change | VIP | Fold_Change |
| Cyanidin O-malonyl-malonylhexoside | - | - | 2.33 | 0.00 | 3.96 | 0.00 |
| Cyanidin O-syringic acid | - | - | 0.84 | 3.38 | - | - |
| Delphinidin | 0.98 | 0.22 | 0.90 | 0.26 | - | - |
| Pelargonidin | 2.68 | up-regulated | 2.70 | up-regulated | 1.40 | 3.11 |
| Cyanidin | - | - | - | - | 1.43 | 3.36 |
| Delphin chloride | - | - | 2.07 | 0.00 | 3.59 | 0.00 |

‘-’ indicates no significant difference in the comparison.

**Table S4** Throughput and quality of *L. japonica* transcriptome data

| Samples^a^ | Raw Reads | Clean Reads | Clean Bases | Raw *Q*30 Bases Rate (%)^b^ | Clean *Q*30 Bases Rate (%) |
| --- | --- | --- | --- | --- | --- |
| GB_Pe_1 | 52,554,608 | 47,372,044 | 7,105,806,600 | 93.74 | 96.71 |
| GB_Pe_2 | 49,904,878 | 45,187,308 | 6,778,096,200 | 93.95 | 96.77 |
| GB_Pe_3 | 50,123,328 | 45,369,930 | 6,805,489,500 | 93.88 | 96.72 |
| WF_Pe_1 | 48,636,608 | 44,364,202 | 6,654,630,300 | 94.17 | 96.78 |
| WF_Pe_2 | 50,602,176 | 46,142,190 | 6,921,328,500 | 94.08 | 96.69 |
| WF_Pe_3 | 50,327,012 | 46,073,580 | 6,911,037,000 | 94.48 | 96.87 |
| YF_Pe_1 | 48,648,046 | 44,428,890 | 6,664,333,500 | 94.3 | 96.81 |
| YF_Pe_2 | 48,964,662 | 45,067,580 | 6,760,137,000 | 94.64 | 96.87 |
| YF_Pe_3 | 48,804,566 | 44,571,092 | 6,685,663,800 | 94.25 | 96.76 |
| Total/Average | 448,565,884 | 408,576,816 | 61,286,522,400 | 94.17 | 96.78 |

^a^1, 2 and 3: Three independent biological replicates

^b^*Q*30: The percentage of bases with a Phred value >30

**Table S5** Summary of *L. japonica* de novo transcriptome assembly

| Header | Transcripts | Unigenes |
| --- | --- | --- |
| Total number | 131,409 | 69,946 |
| Total nucleotides | 139,626,750 | 60,901,270 |
| Percent GC (%) | 39.9 | 39.6 |
| Average length (bp) | 1,063 | 871 |
| Minimum length (bp) | 201 | 201 |
| Maximum length (bp) | 14,346 | 14,346 |
| N50^a^ (bp) | 1,713 | 1,636 |
| N90^b^ (bp) | 463 | 317 |

^a^N50 is defined as the length of the largest contig from all the contigs ranked smallest to largest that represents 50 % of the assembly lengthy.

^b^N90 is defined as the length of the smallest transcript in the sorted list of all transcripts where the cumulative length from the largest transcript to the smallest transcript is at least 90 % of the total length.

**Table S6** Mapping results of *L. japonica* unigenes to various databases

| Database | Count | Percentage | |
| --- | --- | --- | --- |
| BLASTP | 17733 | 25.35 | |
| BLASTX | 23756 | 33.96 | |
| GO | 22662 | 32.4 | |
| KO | 9309 | 13.31 | |
| NR | 31468 | 44.99 | |
| NT | 16799 | 24.02 | |
| PFAM | 18260 | 26.11 | |
| Prot | 23459 | 33.54 | |
| SignalP | 1646 | 2.35 | |
| TmHMM | 5367 | 7.67 | |
| eggNOG | 11867 | 16.97 | |
| Total_anno | 34068 | 48.71 | |
| Total_unigene | 69946 | 100 |  |

^a^N50 is defined as the length of the largest contig from all the contigs ranked smallest to largest that represents 50 % of the assembly lengthy.

^b^N90 is defined as the length of the smallest transcript in the sorted list of all transcripts where the cumulative length from the largest transcript to the smallest transcript is at least 90 % of the total length.

**Table S7** Genes involving in plant hormone transduction and pigments metabolism
